# Variations in the origin, course and branching pattern of dorsalis pedis artery with clinical significance

**DOI:** 10.1038/s41598-020-80555-z

**Published:** 2021-01-14

**Authors:** Hemamalini, H. N. Manjunatha

**Affiliations:** Department of Anatomy, JSS Medical College, JSS AHER, 570015 Mysuru, India

**Keywords:** Anatomy, Health care

## Abstract

Pulsations of the dorsalis pedis artery are commonly used to evaluate the peripheral arterial diseases like thromboangiitis obliterans (TAO) or Buerger’s disease, in lower limbs. Dorsalis pedis artery is a direct extension from the anterior tibial artery and major vascular supply for the dorsum of the foot. But arterial variations in lower limbs are common due to rich distal anastomoses around the ankle joint. Absence of dorsalis pedis arterial pulse does not indicate peripheral arterial disease always as it is sometimes replaced by the enlarged perforating branch of peroneal artery, it may be absent or very thin, deviate laterally on the dorsum of foot. Aim of the present study is to observe the variations in origin, course and branching pattern of dorsalis pedis artery because of its clinical significance. During routine dissection of lower limbs for undergraduates we came across rare variations in the dorsalis pedis artery in its origin, course and branching pattern. Normal anatomic description was found in 27 limbs. In 13 specimens we noted variations, includes bilateral anomalous origin of dorsalis pedis artery, bilateral lateral deviation of dorsalis pedis artery, double dorsalis pedis artery, trifurcation of dorsalis pedis artery and absence of arcuate artery. Knowledge about the arterial variations around the ankle is important to orthopaedic, vascular surgeons and radiologists to prevent complications during surgical interventions.

## Introduction

Popliteal artery is the main artery which supplies the leg and foot by its branches—anterior and posterior tibial arteries. Anterior tibial artery enters anterior compartment through the upper opening in the interosseous membrane and supplies anterior compartment and dorsum of foot. Posterior tibial artery descends down in the posterior compartment and supplies both posterior and lateral compartments of leg and sole of foot. Dorsalis pedis artery begins in front of the ankle joint midway between the malleoli as a continuation of the anterior tibial artery. It runs on the dorsum of foot anteriorly over the dorsal aspect of the talus, navicular and intermediate cuneiform bones till the proximal end of the first dorsal intermetatarsal space and passes inferiorly as the deep plantar artery between the heads of the first dorsal interosseous muscle to complete the plantar arch in the sole of foot with the lateral plantar branch of posterior tibial artery.

### Branches of dorsalis pedis artery

1. Medial and lateral tarsal arteries.
2. Arcuate artery.
3. First dorsal metatarsal artery.
4. Cutaneous branches to the medial side of the dorsum of foot.

The artery may be larger than the normal to compensate small lateral plantar artery. It may be absent^1^. Sometimes an enlarged perforating branch of peroneal artery continues as dorsalis pedis artery^2,3^. There are reports of lateral deviation and bifurcation of the dorsalis pedis artery^4^. The dorsalis pedis artery is best for pedal revascularization since it is the largest artery distal to the ankle joint^5^. The dorsalis pedis flap is one of the most commonly used foot flaps in traffic accidents, electrical burns, industrial injury, ulcer etc. Fasciocutaneous flaps are used for covering defects of the hand^6^.First dorsal metatarsal artery is the most widely used arterial pedicle in toe to finger transplants^7,8^. More recently dorsalis pedis fasciocutaneous flaps are used in the reconstruction of oral cavity defects in cases of oral cancers patients^9^. Osteomyocutaneous peroneal artery perforator flap is being used for reconstruction of the skull base after craniofacial resection in the treatment of malignant cancers which results in the defects of the skull base^10^. In diabetic patients, critical limb ischemia due to occlusions of the lower limb vessels is becoming more common, therefore knowledge of these arteries is needed in order to avoid amputations^11^**.** Aneurysms and pseudoaneurysms of the dorsalis pedis artery are rare vascular complications usually caused by traumatic injury or iatrogenic intervention especially after ankle arthroscopy^12^.

The aim of the study was to see variations in the origin, course and branching pattern of the dorsalis pedis artery.

## Materials and methods

This study was performed on 40 formalin fixed lower limbs of unknown sex in the department of Anatomy, JSS Medical College, Mysuru. The study was conducted during routine dissection for undergraduates as per the Cunningham’s manual for practical anatomy. The compartments of leg and dorsum of foot were dissected along with tracing the anterior tibial artery and dorsalis pedis artery from origin till termination. Variations in the origin, course and branching pattern of dorsalis pedis artery were observed and photographed.

## Results

The normal branching pattern of dorsalis pedis artery was found in 27 limbs out of 40 specimens. Variations observed in 13 limbs were as follows:

### Variation in the origin of dorsalis pedis artery

**Figure 1:**
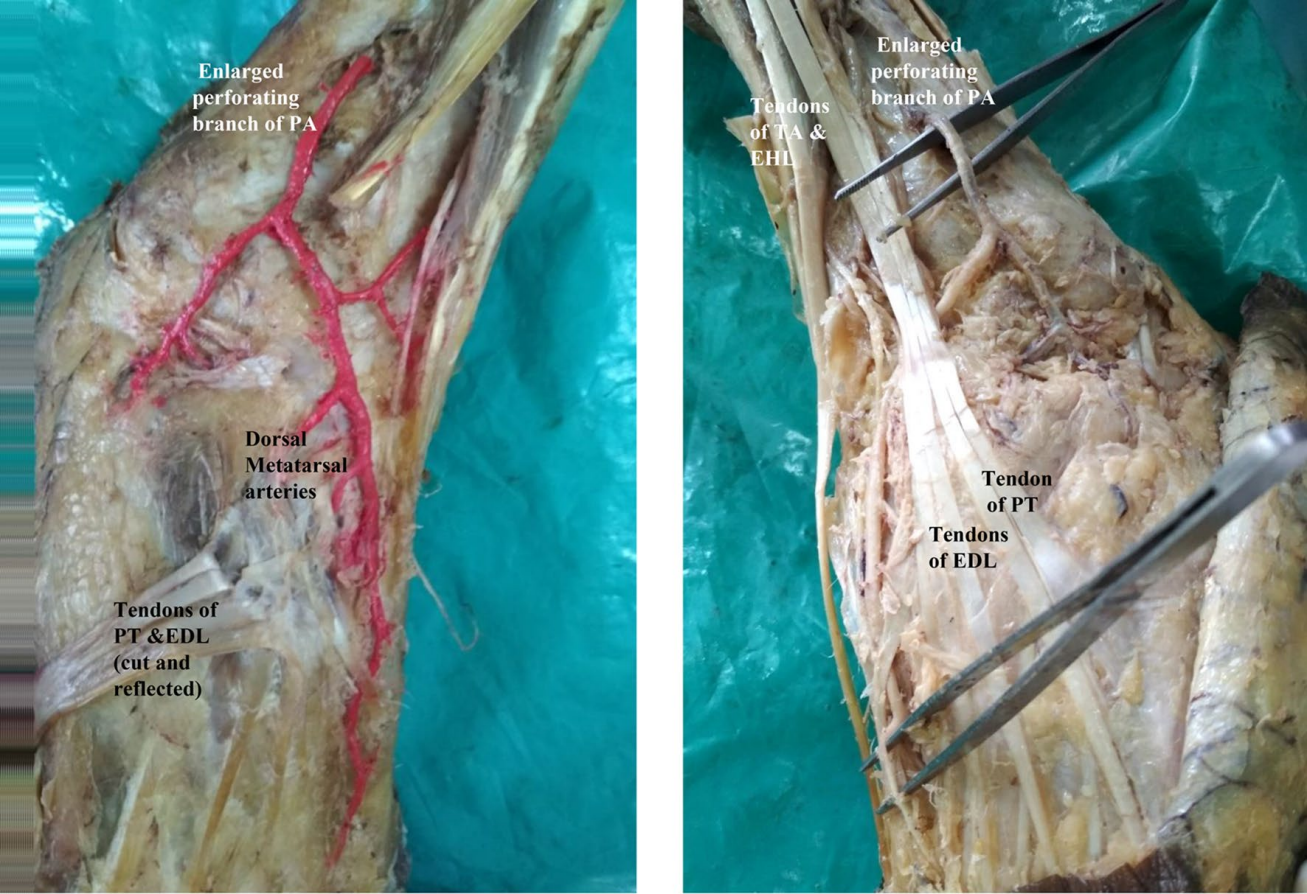
Showing bilateral anomalous origin of dorsalis pedis artery from enlarged perforating branch of peroneal artery and absence of arcuate artery in the right limb and dorsal metatarsal arteries arising from dorsalis pedis artery. TA-Tibialis anterior, EHL-Extensor hallucis longus, EDL-Extensor digitorum longus, PT-Peroneus tertius.

In one specimen, there was bilateral anomalous origin of dorsalis pedis artery from peroneal artery. In both the limbs peroneal arteries were as big as posterior tibial arteries and anterior tibial arteries were smaller in diameter. Anterior tibial artery ended just above the ankle joint. Enlarged perforating branch of peroneal artery after passing through the lower opening in the interosseous membrane, descended anterior to inferior tibiofibular syndesmosis and continued as dorsalis pedis artery. It gave a branch to the lateral side of the dorsum of foot. Main artery runs deep to tendons of peroneus tertius and extensor digitorum longus and then between extensor hallucis longus and extensor digitorum longus and gave first dorsal metatarsal artery and entered sole as deep plantar artery (Fig. 1).

**Figure 2:**
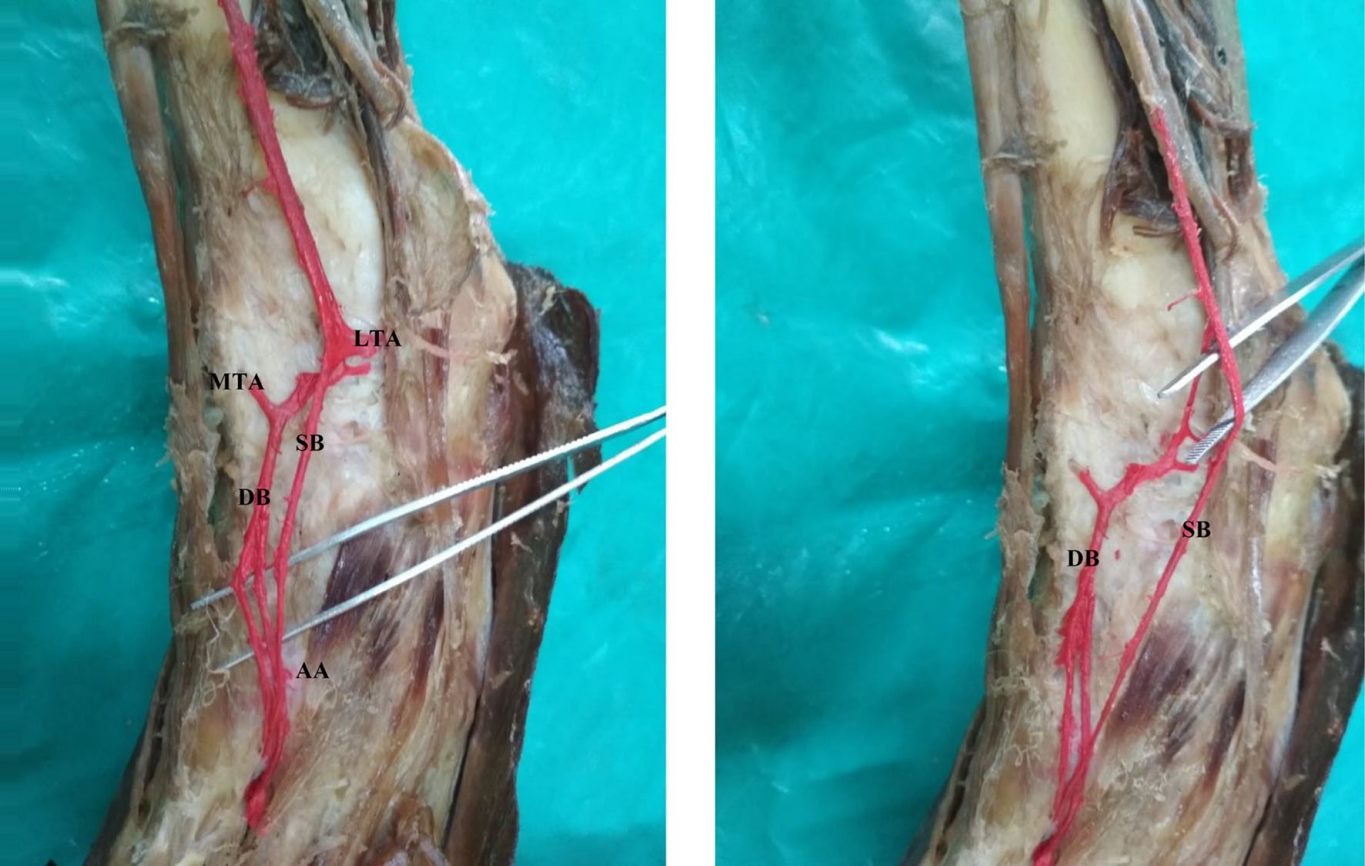
Showing double dorsalis pedis artery. SB-superficial branch, DB-deep branch, LTA-lateral tarsal arteries, MTA-medial tarsal artery, AA-arcuate artery.

In another specimen, anterior tibial artery divided into two branches (superficial and deep) above the talocrural joint, and both branches entered dorsum as dorsalis pedis arteries—double dorsalis pedis artery. The deep branch gave one more branch and all three ran side by side to reach the first dorsal intermetatarsal space. The middle one dipped into the first dorsal metatarsal space as deep plantar artery and gave the first dorsal metatarsal artery (Fig. 2).

### Variation in the course of dorsalis pedis artery

**Figure 3:**
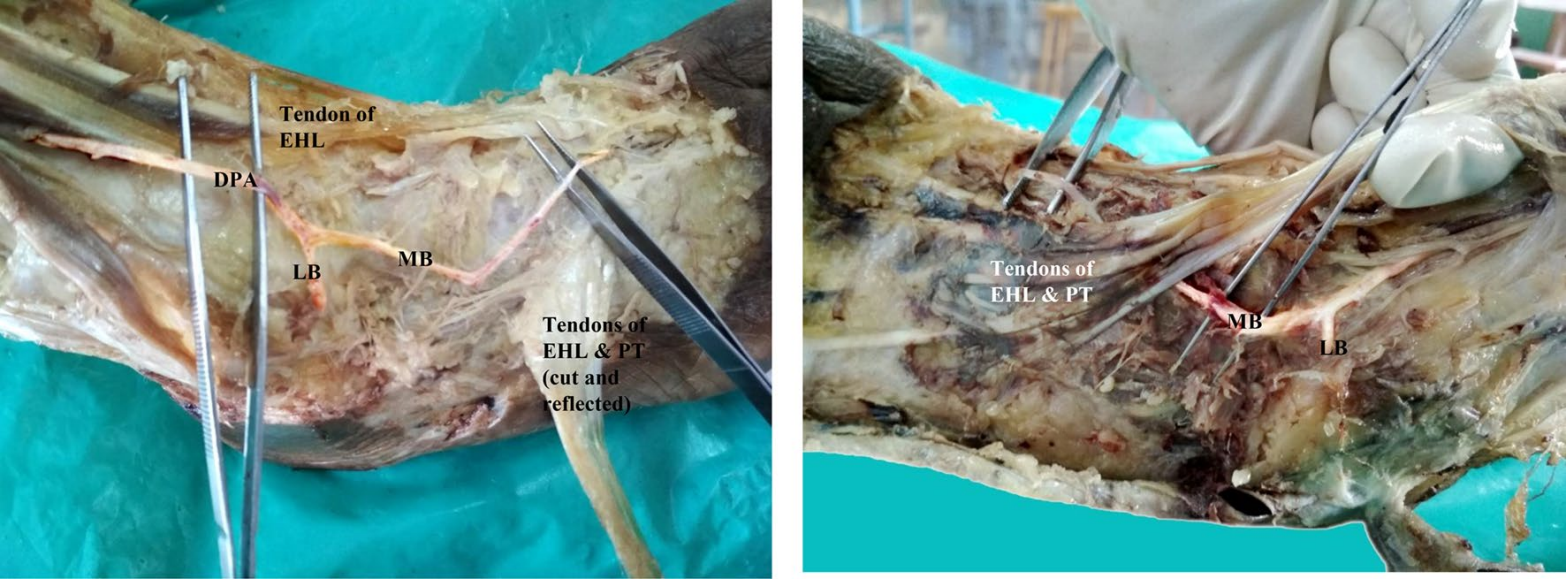
Showing bilateral deviation and bifurcation of dorsalis pedis artery and extremely thin dorsalis pedis artery in right limb. DPA-Dorsalis pedis artery, LB-lateral branch, MB-medial branch.

In another specimen, there was bilateral deviation towards lateral side and it divided into two branches. The medial branch continued as dorsalis pedis artery passing deep to the tendons of peroneus tertius and extensor digitorum longus and gave first dorsal metatarsal artery and entered sole as deep plantar artery. But the medial branch in right limb was extremely thin, could not be traced beyond the first dorsal metatarsal space, when compared to the left limb (Fig. 3).

**Figure 4:**
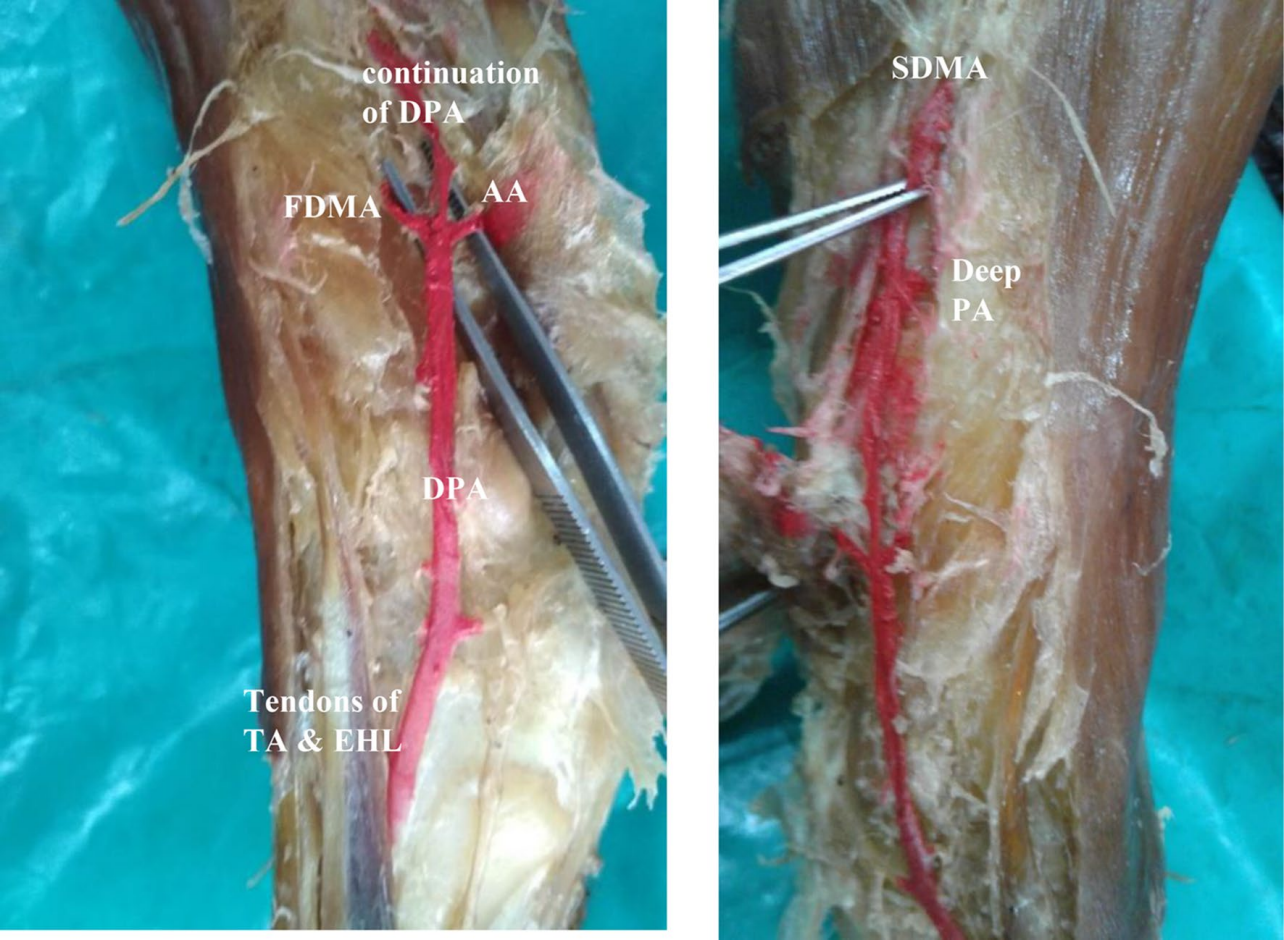
Showing trifurcation of dorsalis pedis artery and entering sole in the II inter metatarsal space. FDMA-first dorsal metatarsal artery, AA-arcuate artery, continuation of DPA – continuation of dorsalis pedis artery, Deep PA-deep plantar artery, SDMA-second dorsal metatarsal artery.

In one specimen, the dorsalis pedis artery was trifurcating into arcuate artery on the lateral side, first dorsal metatarsal artery on the medial side and middle one continued as dorsalis pedis artery just distal to ankle joint. Traced distally, the dorsalis pedis artery, gave second dorsal metatarsal artery and deep plantar artery and entered sole in the second dorsal inter metatarsal space instead of first space (Fig. 4).

### Variation in the branching pattern of dorsalis pedis artery

**Figure 5:**
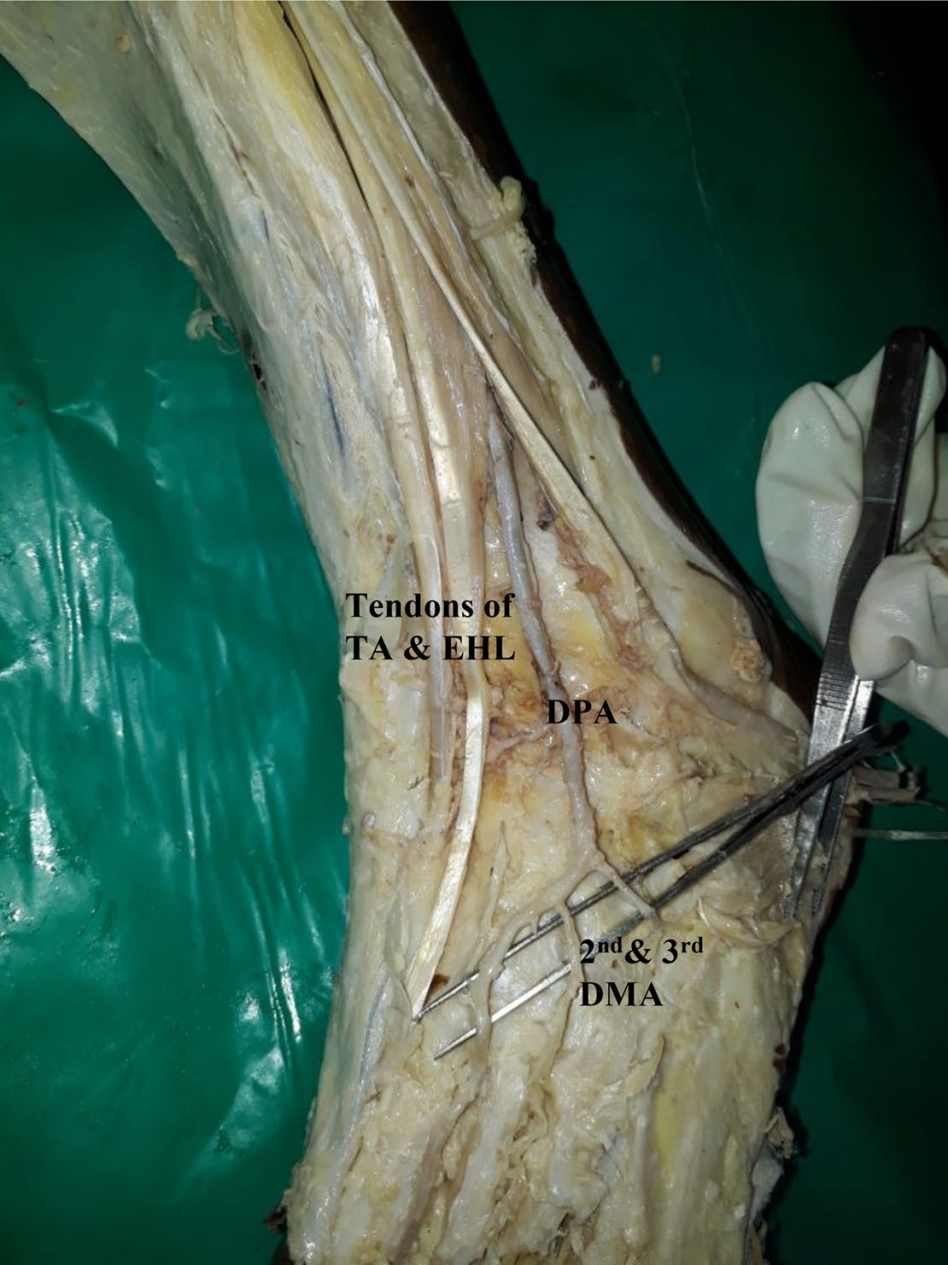
Showing 2nd and 3rd dorsal metatarsal arteries arising directly from the dorsalis pedis artery. DMA-Dorsal metatarsal arteries.

**Figure 6:**
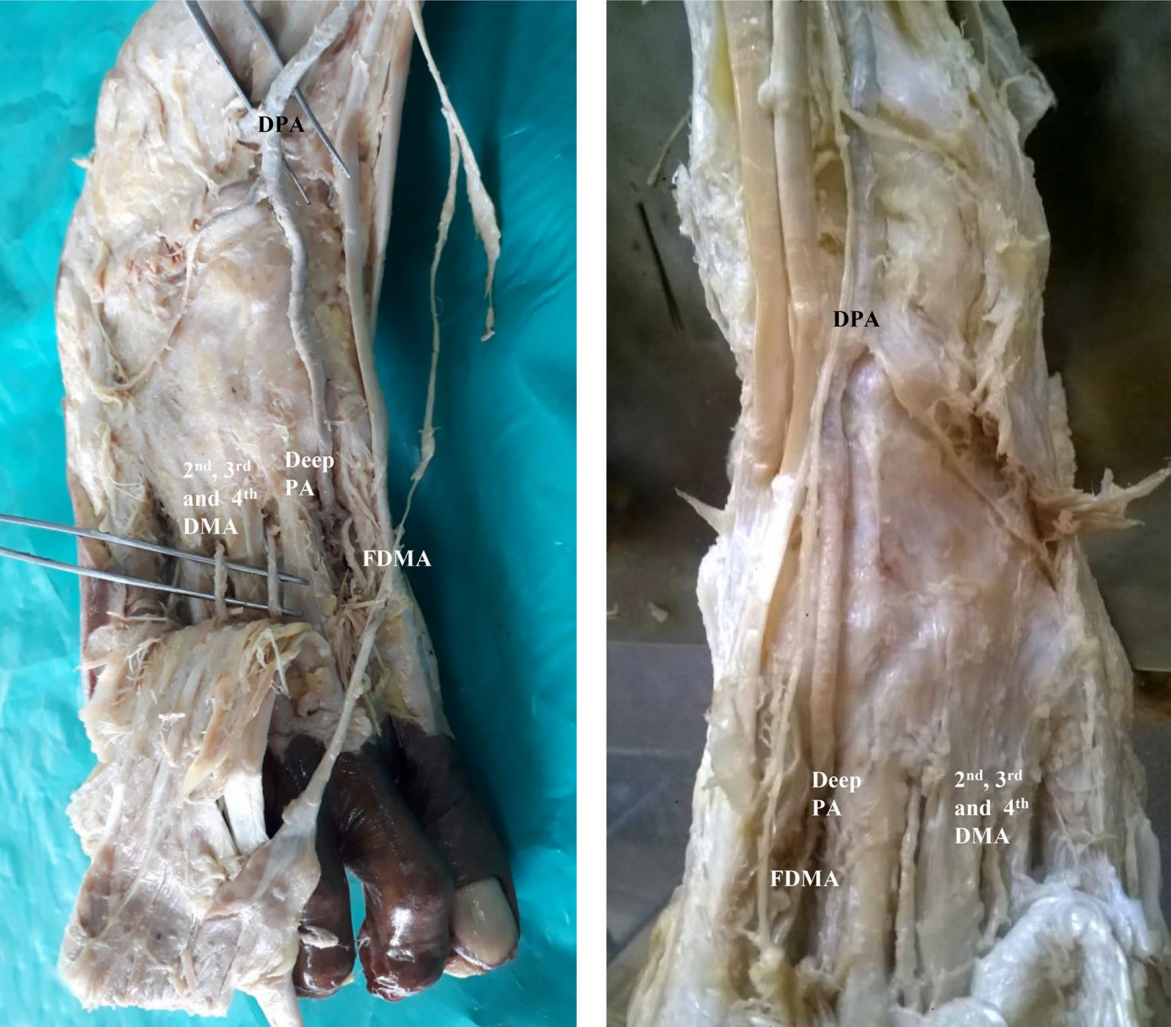
Absence of arcuate artery with dorsal metatarsal arteries arising from plantar arch. FDMA-First dorsal metatarsal artery, Deep PA-Deep plantar artery.

**Figure 7:**
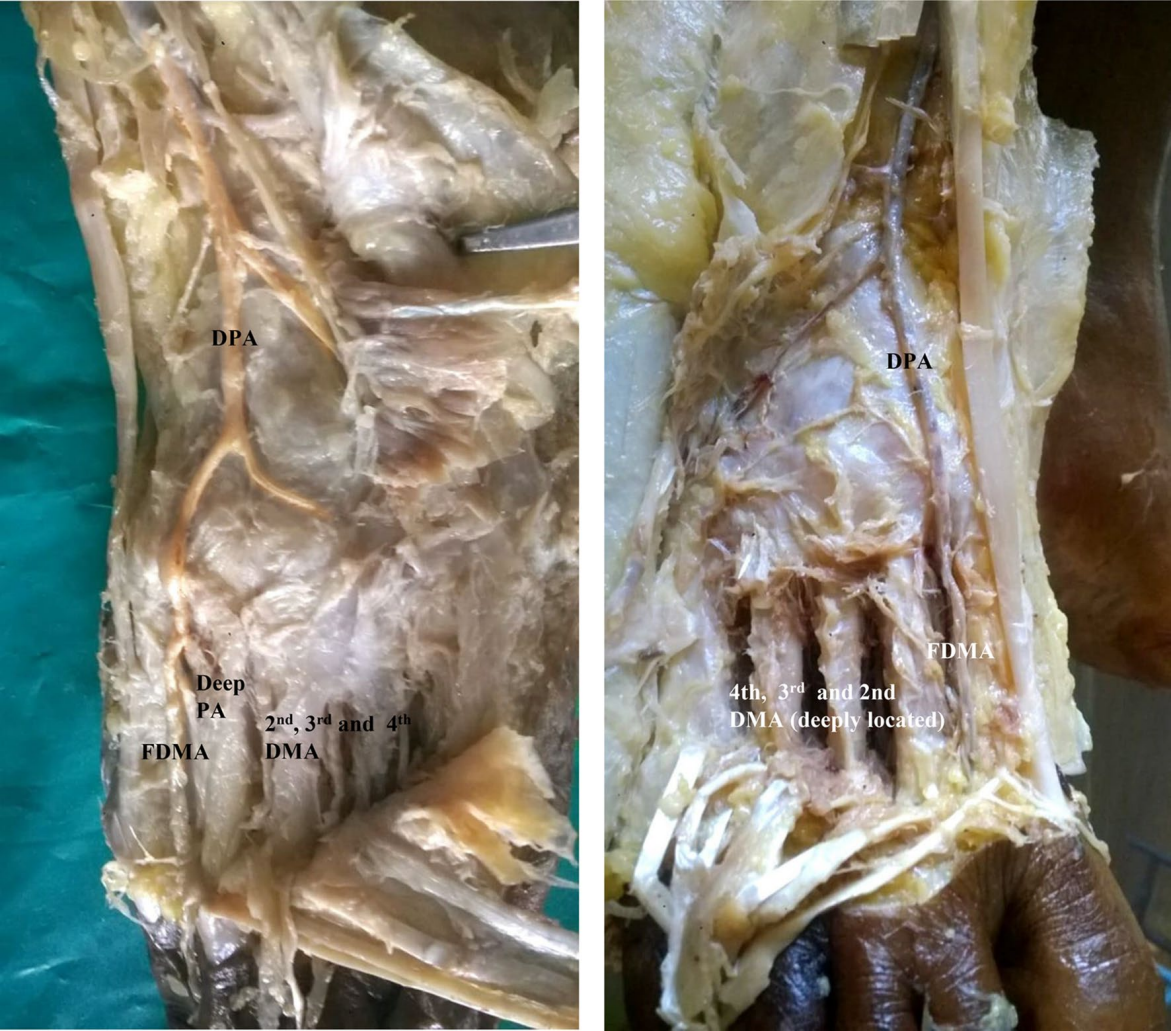
Dorsal metatarsal arteries arising from plantar arch. FDMA-First dorsal metatarsal artery, DMA-Dorsal metatarsal arteries.

**Figure 8:**
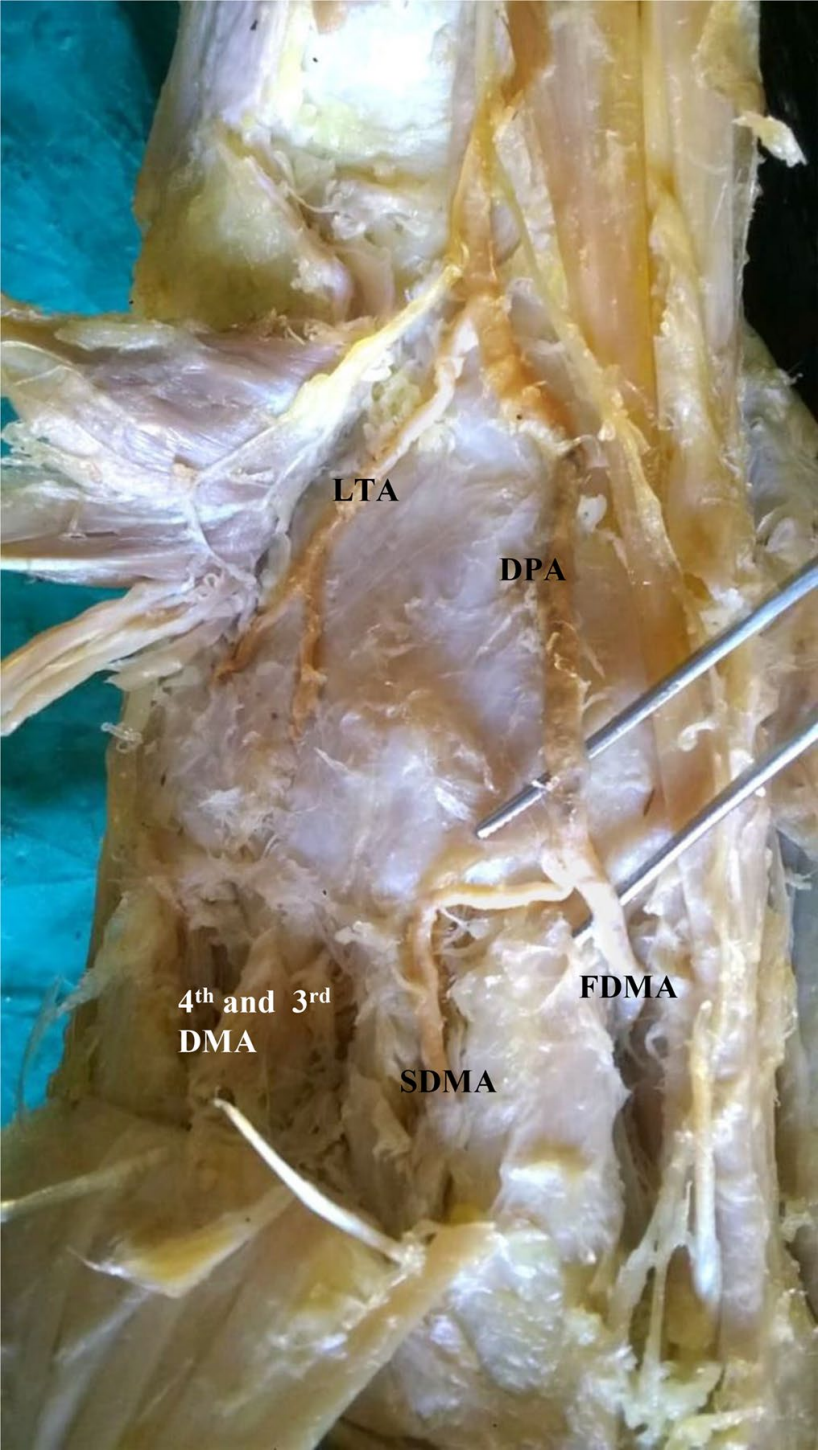
Absence of arcuate artery with 2nd dorsal metatarsal artery arising from dorsalis pedis artery, 3rd and 4th dorsal metatarsal arteries from plantar arch. SDMA-Second dorsal metatarsal artery, LTA-lateral tarsal artery.

**Figure 9:**
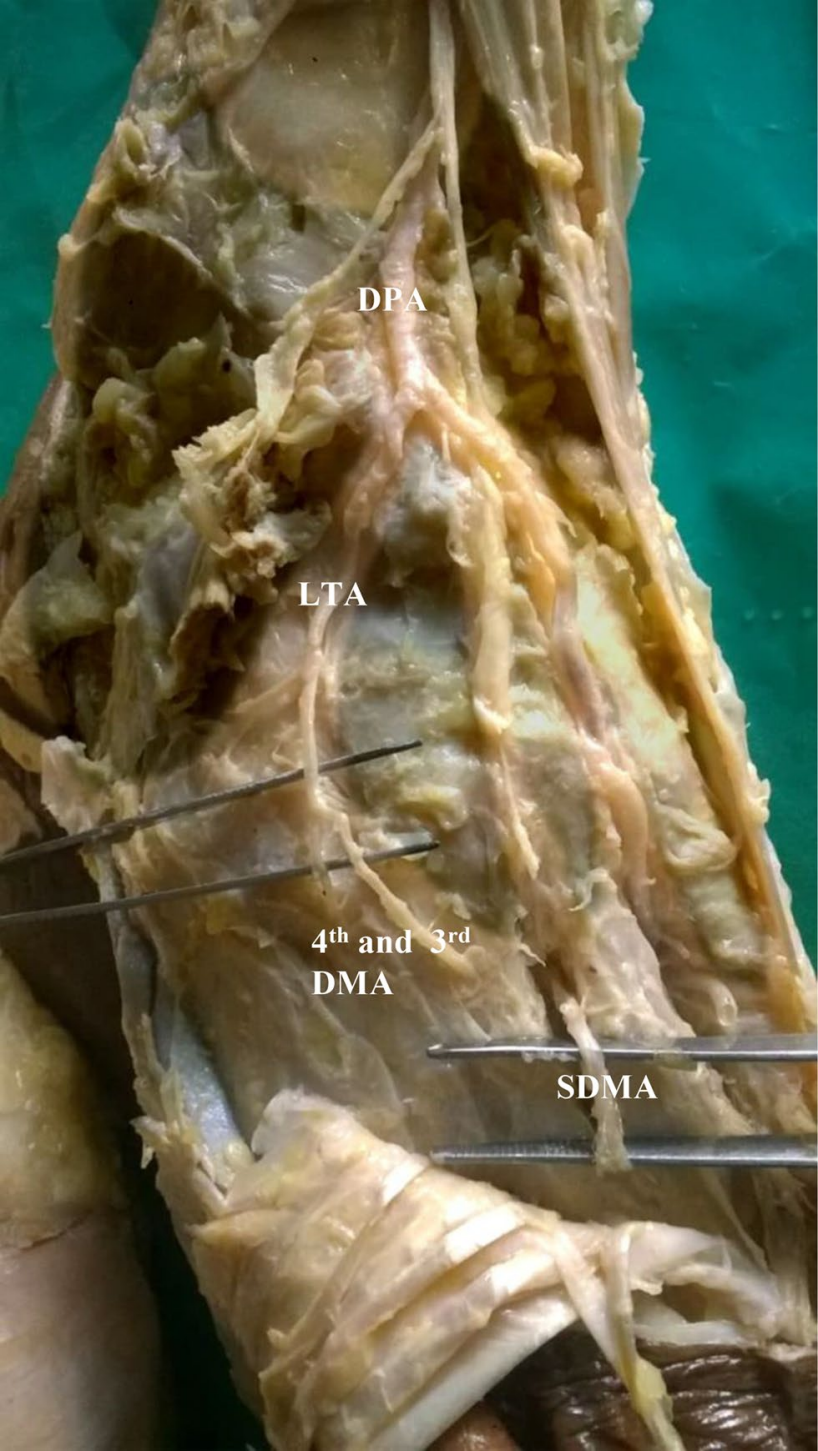
Absence of arcuate artery with 2nd dorsal metatarsal artery arising from plantar arch, 3rd and 4th dorsal metatarsal arteries from lateral tarsal artery.

In seven specimens, arcuate artery was absent. Since arcuate artery was absent, there were variations in the origin of dorsal metatarsal arteries.

(i). In the same right anomalous origin of dorsalis pedis artery by enlarged perforating branch of peroneal artery specimen, there was absence of arcuate artery. The 2nd, 3rd and 4th dorsal metatarsal arteries were taking origin from the dorsalis pedis artery directly.
(ii). In one specimen, 2nd and 3rd dorsal metatarsal arteries arising directly from dorsalis pedis artery (Fig. 5).
(iii). The 2nd, 3rd and 4rd dorsal metatarsal arteries arising from the plantar arch in four specimens (Figs. 6 and 7)
(iv). Dorsalis pedis artery was slightly tortuous and giving rise to 2nd dorsal metatarsal artery (Fig. 8).
(v). The 2nd dorsal metatarsal artery from plantar arch, 3rd and 4th dorsal metatarsal arteries from lateral plantar artery (Fig. 9).

## Discussion

Abnormal or variations in the branching patterns of the popliteal artery results due to arrest of normal development of limb vessels according to Senior^13^. The lower limb vessels arise from sciatic artery, a branch of the umbilical artery. Sciatic artery persists in most vertebrates, but in mammals the femoral artery, as the continuation of the external iliac artery, becomes the main artery for the lower limbs. Anastomoses are formed between the sciatic and the femoral arteries. When the femoral artery continues as the popliteal artery, the sciatic artery regresses. Its middle and distal portions persist to form the popliteal and peroneal arteries, whereas the anterior tibial and posterior tibial arteries are derived from the femoral artery. So regression and persistence of the sciatic artery and its connection with the femoral artery in the popliteal region are the main reasons for the variations in the arteries of the leg^14^.

Hypoplasia of one of these vessels can cause abnormal blood supply to the foot^15^. Tuncel et al., reported that in 8% of cases the dorsalis pedis artery was replaced by peroneal artery^2^. Cheung et al., also reported abnormal origin of dorsalis pedis artery from peroneal artery^3^.

But according to Yamada et al., the dorsalis pedis artery arise from peroneal artery in 6.7% of cases^16^. In a study by Vijayalakshmi et al., the dorsalis pedis artery was found to have a normal course and branching pattern in 56%, variation in origin in 8%, variation in branching pattern in 16% of cases^17^. In present study, there was bilateral anomalous origin of dorsalis pedis artery by the peroneal artery. Similar anomalous origin was described bilaterally by Tuncel et al.^2^, and unilaterally by Ali et al.,^18^. Vijayalakshmi et al., reported double dorsalis pedis, which continued as first dorsal metatarsal artery and deep plantar artery respectively^19^. But in the present study, there were three arteries running parallel to each other and the middle one continued as deep plantar artery after giving first dorsal metatarsal artery. Both superficial and deep arteries gave lateral tarsal arteries. But medial tarsal was arising from the deep branch and arcuate artery from the superficial branch. This type of variation has not been reported till now in the literature.

Lateral deviation and bifurcation of dorsalis pedis artery has been reported by Bailleul et al., in their study just 2–3 cm distal to its origin^4^. In the present study also dorsalis pedis artery showed bifurcation just distal to ankle joint, the medial branch continued as dorsalis pedis artery after passing deep to the tendons of peroneus tertius and extensor digitorum longus and returned to the first dorsal inter metatarsal space in the distal part of the foot.

In another study by Kesavi et al., reported that the most favorable point for anastomosis of dorsalis pedis artery on the foot is the segment distal to the ankle^20^. If the course of dorsalis pedis artery is oblique it may result in impaired healing in fracture of distal end of tibia, fibula and malleoli due to external or internal fixation^3^. Anomalous origin of dorsalis pedis artery like this and lateral deviation of dorsalis pedis artery passing anterior to inferior tibiofibular syndesmosis may get damaged during ankle arthroscopy.

Trifurcation of dorsalis pedis artery is a rare variation not reported so far in the literature. Lateral branch was arcuate artery, medial branch was first dorsal metatarsal artery and middle one continued as dorsalis pedis artery. After giving second dorsal metatarsal artery it entered sole as deep plantar artery in the second dorsal inter metatarsal space instead of first dorsal intermetatarsal space. This variation is also not mentioned in the literature. However, Nilesh et al., have reported the origin of II dorsal metatarsal artery from dorsalis pedis artery, in case of absence of arcuate artery^21^. But in the present case, second dorsal metatarsal artery was arising from dorsalis pedis artery even in the presence of arcuate artery is not reported in the literature.

In the present study, we also got another not described variation, that is, in the absence of arcuate artery the 2nd 3rd and 4th dorsal metatarsal arteries were arising from dorsalis pedis artery and not from the lateral tarsal artery.

According to Nilesh et al., the 3rd and 4th dorsal metatarsal arteries arise from lateral tarsal artery if arcuate artery is absent and 2nd dorsal metatarsal artery from dorsalis pedis artery. But according to DiLandro et al., the arcuate artery is not the main arterial supply to dorsal metatarsal arteries as it is commonly described since the arcuate artery was present only in 16.7% of 72 specimens they studied. It was the lateral tarsal artery which gave dorsal metatarsal (2nd, 3rd and 4th) arteries more frequently (47.2%) than the arcuate artery^22^.

In four specimens, the 2nd, 3rd and 4th dorsal metatarsal arteries were arising from plantar arch, in the absence of arcuate artery, which is the most common source for the origin of dorsal metatarsal arteries accounting for 40% according to Bergman’s Comprehensive Encyclopedia of Human Anatomic Variation and the arcuate artery gives origin to dorsal metatarsal arteries only in 20% of cases^23^.

We are reporting three significant variations not reported in the literature are—in the dorsalis pedis artery origin (Double dorsalis pedis and it termination) branching pattern (2nd, 3rd and 4th dorsal metatarsal arteries arising from dorsalis pedis artery in the absence of arcuate artery and not from lateral tarsal artery) and termination (trifurcation of dorsalis pedis artery and entering the sole in the second dorsal metatarsal space instead of first dorsal metatarsal space)as it is important for radiologists, vascular surgeons and reconstructive surgeons in order to prevent complications like ischemia and subsequent necrosis due to vascular trauma. Table 1 showing compilation of the previous studies with present study.

**Table 1:** Showing compilation of the data available on the variations of dorsalis pedis artery in origin, course and branching pattern published in the literature till date and comparing them with the present study variations.

Author name and year	No of cases	Normal pattern	Replaced by peroneal artery	Dorsalis pedis artery absent	Absent arcuate artery	Lateral deviation	Duplication (double dorsalis pedis artery	Divided into medial and lateral branches	Ending in second metatarsal space	Variations in the origin of dorsal metatarsal arteries
Bailleul^4^	67	65.6%	–	8.9%	–	–	–	22.3%	2.9%	
Yamada^16^	30	53.3%	6.7%	6.7%	33%	–	–	–	–	
Ebrahim^24^	20	–	–	–	10%	5%	–	–	–	
Vijayalakshmi^17^	50	56%	8%	2%	6%	–	2%	–	–	
Vasudha^25^	33	15%	12.1%	9.1%	–	6%	–	–	–	
Rajeshwari^26^	40	54.7%	–	9.52%	16.6%	–	–	–	–	
SharadKumar^27^	100	96%	–	–	–	–	–	4%	–	
Mamatha^28^	–		–	2%	6%	–	–	16%	–	
Sanjay^29^	50	98%	2%	–	–	–	–	–	–	
Mamta^30^	40	72.5%	7.5%	2.5%	5%	7.5%	–	–	–	
Present study	40	67.5%	5% Bilateral	–	17.5%*	5% Bilateral	2.5% 1 case	–	2.5% 1 case	17.5%

*In the same specimen where dorsalis pedis artery was replaced by peroneal artery right limb, arcuate artery was absent in the right limb).

## Conclusion

Variation in the origin course and branching pattern of lower limb arteries are accidently found during dissections of the foot. Precise knowledge of this is important for surgeons and radiologists who operate and do interventional procedures in this area.Retrograde pedal access is a viable revascularization technique for saving limb in patients with critical limb ischemia. Revascularization is the main therapy for restoring adequate blood supply to the wound, to promote healing in diabetic foot, thus avoiding major amputations. First dorsal metatarsal artery is the most widely arterial pedicle used in toe to hand transplants. Success of the transplant depends on the diameter of the first dorsal metatarsal artery and not the space. As variation in dorsalis pedis artery is quite common, it is essential to have a preoperative angiography for any abnormality, to prevent risks during surgical intervention.
